# Mitochondrial Implications in Cardiovascular Aging and Diseases: The Specific Role of Mitochondrial Dynamics and Shifts

**DOI:** 10.3390/ijms23062951

**Published:** 2022-03-09

**Authors:** Anastasia V. Poznyak, Tatiana V. Kirichenko, Evgeny E. Borisov, Nikolay K. Shakhpazyan, Andrey G. Kartuesov, Alexander N. Orekhov

**Affiliations:** 1 Institute for Atherosclerosis Research, 121609 Moscow, Russia; omi@bk.ru; 2 National Medical Research Center of Cardiology, Institute of Experimental Cardiology, 121552 Moscow, Russia; 3 Laboratory of Angiopathology, Institute of General Pathology and Pathophysiology, Russian Academy of Medical Sciences, 125315 Moscow, Russia; andkartuesv@gmail.com; 4 AP Avtsyn Research Institute of Human Morphology, 117418 Moscow, Russia; borisovevgenij5@gmail.com (E.E.B.); nshakhpazyan@gmail.com (N.K.S.)

**Keywords:** atherosclerosis, ischemic stroke, cardiovascular disease, mitochondria, mitochondrial dynamics

## Abstract

Cardiovascular disease has been, and remains, one of the leading causes of death in the modern world. The elderly are a particularly vulnerable group. The aging of the body is inevitably accompanied by the aging of all its systems, and the cardiovascular system is no exception. The aging of the cardiovascular system is a significant risk factor for the development of various diseases and pathologies, from atherosclerosis to ischemic stroke. Mitochondria, being the main supplier of energy necessary for the normal functioning of cells, play an important role in the proper functioning of the cardiovascular system. The functioning of each individual cell and the organism as a whole depends on their number, structure, and performance, as well as the correct operation of the system in removing non-functional mitochondria. In this review, we examine the role of mitochondria in the aging of the cardiovascular system, as well as in diseases (for example, atherosclerosis and ischemic stroke). We pay special attention to changes in mitochondrial dynamics since the shift in the balance between fission and fusion is one of the main factors associated with various cardiovascular pathologies.

## 1. Introduction

Mitochondria are the a main energetic organelles of the cell. They are responsible for the production of the main part of the energy utilized for cells’ metabolism. Mitochondria are also required for Ca2+ homeostasis, autophagy, apoptosis, and other essential cellular processes [1]. There are two membranes in mitochondria: the outer mitochondrial membrane (OMM) and the inner mitochondrial membrane (IMM), while the space between these two membranes is referred to as the intermembrane space. These membrane structures mostly consist of phospholipids, but the distribution and the lipid composition differ in IMM and OMM [2]. These molecules are a key element of the protein transport into the mitochondria and also play an important role in the general dynamics and functioning of mitochondria. Phospholipids are produced in the endoplasmic reticulum and then delivered to the mitochondria [3]. Thus, violations of the composition of phospholipids can alter numerous mitochondrial characteristics, including membrane integrity, fluidity, and permeability. This results in problems in the stability and activity of several proteins associated with the inner membrane, including those involved in electron transport chains and oxidative phosphorylation [4].

Numerous human diseases, including cardiovascular pathologies, are based, at least in part, on mitochondrial dysfunction. This dysfunction leads to oxidative stress, which arises from the over-production of reactive oxygen species (ROS) by the mitochondria as by-products of the respiratory chain functioning [5]. Normally, ROS act as messenger molecules, and their levels are low. The overproduction of ROS and their overcoming the antioxidant capability of cells result in pathology development via oxidative damage to various biomolecules. This means that dysfunctional mitochondria are potential sites of uncontrolled ROS production and cell damage [6].

Today’s knowledge suggests that mitochondria are derived from prokaryotic endosymbionts. This hypothesis is supported by the fact that they share a certain degree of autonomy and some characteristic features of bacteria [7]. Such characteristics include circular mitochondrial DNA (mtDNA), which can exist in many copies, as well as bacterial chromosomes. Mutations of mtDNA are usually described at the heteroplasmy level or as a percentage of mutant copies per genome [4].

## 2. Mitochondrial Dynamics

### 2.1. Mitochondrial Fusion

**Figure 1:**
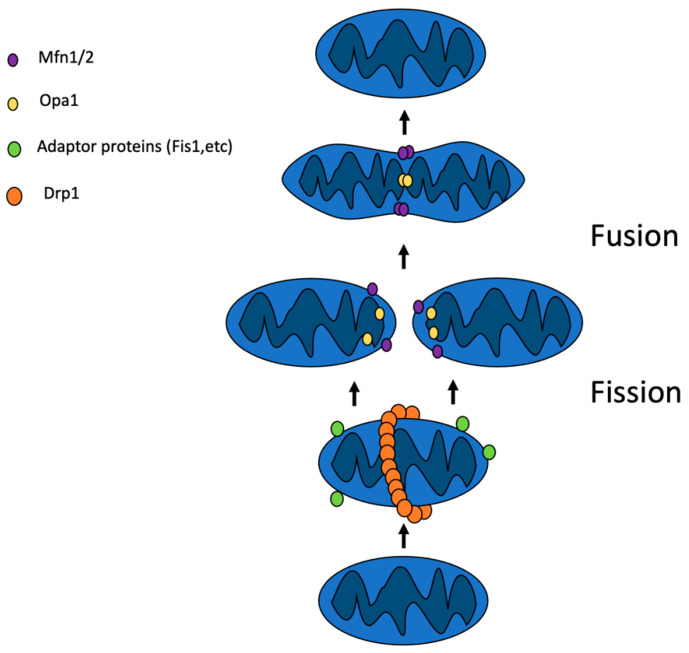
Mitochondrial dynamics. Fusion is controlled by Mfn1 (mitofusin1), Mfn2 (mitofusin 2), and Opa1 (optic atrophy protein 1); fission is mediated by Drp1 (dynamin-related protein 1), and adaptor proteins, such as Fis1 (mitochondrial fission protein 1), and others.

Mitochondrial fusion is the process of merging of mitochondrial membranes of two separate organelles. During the process of mitochondrial fusion, both of the mitochondrial membranes of the consolidating organelles merge (see Figure 1). This process is controlled by a range of proteins, such as mitofusin 1 (Mfn1), mitofusin 2 (Mfn2), and optic atrophy protein 1 (Opa1), all of which belong to the dynamin-related GTPases family [8]. The fusion of OMM is mediated by mitofusins 1 and 2, while the fusion of the inner mitochondrial membranes requires the presence of optic atrophy protein 1. Mfn 1 and Mfn 2 isoforms are able to compensate for the lack of the other in the case of a lowered expression of one of them. During the fusion, Mfn1/2 connect to their homologues, which are located at the OMM of the other participating mitochondrion, which is followed by the power stroke generated by guanosine triphosphate (GTP) hydrolyses. This is crucial for membrane fusion [9] (see Table 1).

Opa1 GTPase can be found in the IMM, and this protein is required not only for mitochondrial fusion, but also for cristae remodeling due to mitochondrial damage or energetic stress. This contributes to the IMM retaining its integrity and its function [10]. Opa1 undergoes proteolytic cleavage, changing from a long-anchored isoform (L-Opa1) to a short one (S-Opa1). Oligomerization of an S-Opa1 isoform with two L-Opa1 subunits results in a complex tightening of the cristae and the stimulation of oxidative phosphorylation by increasing the ATP synthase assembly. It is important to note that Opa1 mutants that maintain the ability to oligomerize can also sustain the cristae structure, but exert no fusion activity [11].

Various proteins mediate the Opa1 proteolytic cleavage: paraplegin, presenilin-associated rhomboid-like (Parl) protein, mitochondrial metalloendopeptidase Oma1, ATP-dependent zinc metalloprotease YME1 Like 1 ATPase (Yme1L1), and mAAA protease complex ATPase family gene-3 yeast-like-1 (Afg3l1) [12]. The acetylation of lysine residues is an important post-translational modification controlling Opa1 activity. Cardiac stress contributes to the increased Opa1 hyperacetylation linked to the decreased activity of GTPase. Sirtuin 3, which is known for its beneficial effects on mitochondrial metabolic enzymes, can reverse this process by direct binding to Opa1 and stimulating mitochondrial function and dynamic networking [13].

L-Opa1 components are crucial for the maintenance of mitochondrial respiration and for mitochondrial fusion. Thus, the enhanced production of S-Opa1 is due to the elevated activity of Oma1. This leads to the accumulation of S-Opa1 in the intermembrane space, which, in turn, moves mitochondrial dynamics to the fission side [14].

### 2.2. Mitochondrial Fission

Mitochondrial fission is the process of dividing mitochondria into two separate organelles (see Figure 1). The main regulator of mitochondrial fission is dynamin-related protein 1 (Drp1). Interestingly, in the primary sequence of Drp1, no mitochondrial targeting motifs were found. However, after activation, Drp1 moves from the cytosol to mitochondria [15]. This translocation is possible due to the activity of adaptor proteins located in the OMMs, such as mitochondrial fission protein 1 (Fis1), mitochondrial division protein 1 (Mdv1), and mitochondrial fission factor (Mff) [16]. After the recruitment to the OMM, Drp1 undergoes GTP-dependent oligomerization forming a spiral ring with an inner diameter of 20 nm [17] (see Table 1).

**Table 1:** Proteins responsible for mitochondrial dynamic processes.

Protein	Process	Role in Dynamics	Reference
Mfn1	Fusion	Fusion of OMM	[9]
Mfn2	Fusion	Fusion of OMM	[9]
Opa1	Fusion	Fusion of IMM	[9]
Paraplegin	Fusion	Mediates the Opa1 proteolytic cleavage	[10]
Parl	Fusion	Mediates the Opa1 proteolytic cleavage	[10]
Oma1	Fusion	Mediates the Opa1 proteolytic cleavage	[10]
Yme1L1	Fusion	Mediates the Opa1 proteolytic cleavage	[10]
Afg3l1	Fusion	Mediates the Opa1 proteolytic cleavage	[10]
Drp1	Fission	Forms a ring around the mitochondrion, separating it in two	[16]
Fis1	Fission	Adaptor protein located in the OMM	[16]
Mdv1	Fission	Adaptor protein located in the OMM	[16]
Mff	Fission	Adaptor protein located in the OMM	[16]
dynamin 2	Fission	Mediates membrane reorganization	[18]
endophilin 1	Fission	Mediates membrane reorganization	[18]
SNX9	Fission	Mediates membrane reorganization	[18]

Mfn1—mitofusin1; Mfn2—mitoofusin 2; Opa1—optic atrophy protein 1; Parl—presenilin-associated rhomboid-like protein; Oma1—mitochondrial metalloendopeptidase Oma1; Yme1L1—ATP-dependent zinc metalloprotease YME1 Like 1 ATPase; Afg3l1—mAAA protease complex ATPase family gene-3 yeast-like-1; Drp1—dynamin-related protein 1; Fis1—mitochondrial fission protein 1; Mdv1—mitochondrial division protein 1; Mff—mitochondrial fission factor; SNX9—sorting nexin 9; OMM—outer mitochondrial membrane; IMM—inner mitochondrial membrane.

Mitochondrial fission involves the tubing of all four thick juxtaposed mitochondrial membranes into a narrow lumen. This stimulates a reorganization involving dynamin 2, endophilin 1, and sorting nexin 9 (SNX9) [18]. The activity of Drp1, such as enrollment and oligomerization, can be managed by phosphorylation, S-nitrosylation, ubiquitination, sumoylation, and other post-translational modifications. This is illustrated by the protein kinase A, which suppress the activity of Drp1, and the kinase Cdk1/cyclin B, which enhances the early stress response, including during mitosis [19]. Phosphorylation at serine 637 of Drp1 by serine/threonine kinase Pim1 was shown to suppress the activity of Drp1. Conversely, calcineurin dephosphorylates Drp1, thus promoting its activity. Proteasomal degradation of Drp1 can be promoted by ubiquitination by the E3-ubiquitin ligase, parkin, which negatively regulates Drp1 [20]. Drp1 also undergoes sumoylation, a post-translation modification that enhances its activity. Mitochondrial-anchored protein ligase (MAPL), a small ubiquitin-like modifier ligase (SUMO)-3, was also identified as enhancing mitochondrial fission. However, some investigations on different members of the sentrin-specific protease (SENP) family, which reverse SUMO conjugation in mammalian cells, led to controversial results. SENP-3-desumoylated Drp1 and reduced Drp1 were reported to enhance cell death in ischemic conditions. Additionally, cardiac overexpression of SENP5 stimulated apoptosis and cardiomyopathy in murine models [21,22].

Drp1 plays an important role in mitophagy and mitochondrial permeabilization regulation, thereby promoting the intrinsic apoptotic pathway. Colocalization of Drp1 and the proapoptotic factor Bcl-2-associated X-protein (Bax) was also reported, as well as inhibition of apoptosis and fission in response to Bax downregulation [23]. On the other hand, several studies have shown no linkage between Bax-induced apoptosis and fission activation. Additionally, Bcl-xL overexpression was reported to block apoptosis without inhibiting mitochondrial fission [24].

### 2.3. Mitochondrial Dynamics and Mitophagy Interplay

**Figure 2:**
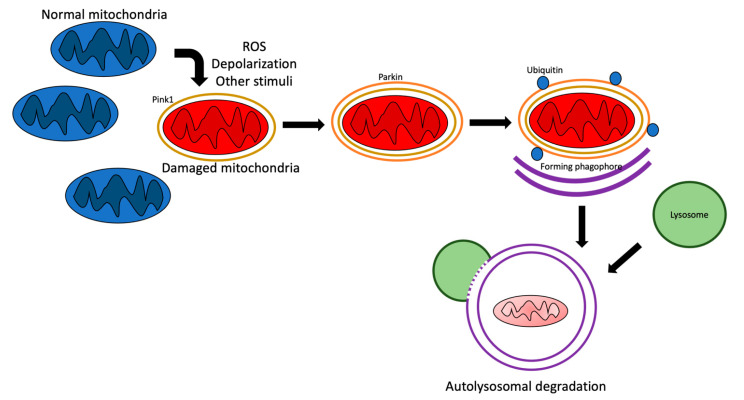
Schematic representation of the mitophagy process. ROS—reactive oxygen species.

Mitophagy (see the schematic representation in Figure 2) is the specific autophagy form, the action of which consists of damaged or senescent mitochondria digestion. Mitophagy can be realized via two mechanisms, one of which depends on parkin. In the parkin-dependent mechanism, the phosphatase and tensin homologue (PTEN)-induced kinase 1 (PINK1) recruits parkin in the OMM [25]. Direct phosphorylation of parkin and indirect mechanisms, such as Mfn2 phosphorylation by PINK1, is recognized by parkin, which subsequently localizes to the mitochondria [26]. These data contribute to the existing interaction of mitophagy and mitochondrial dynamics. After parkin activation, various targets may be ubiquitinated, which promotes their interaction with mitophagy adaptors, for example, p62/sequestosome 1. This is followed by the interaction between the recruited sequestosome 1 with microtubule-associated protein 1 light chain 3 (LC3), which mediates an uptake of the damaged mitochondria into autophagosomes [27]. Then, the engulfed mitochondria undergo digestion within the lysosomes fusing with the autophagosomes, which form so-called ‘autolysosomes’. However, there are several mechanisms, such as the suppression of parkin translocation to OMM by p53 and the deubiquitination of its targets by USP15 and USP30 on outer mitochondrial membranes proteins, which counteract the activity of parkin [28].

Additionally, mitophagy can be mediated by parkin-independent mechanisms. Crucial proteins for these mechanisms include Bcl2/adenovirus E1B 19-kDa protein-interacting protein 3 (Bnip3), Bcl2-like protein 13 (Bcl2-L-13), cardiolipin, FUN14 domain-containing protein 1 (Fundc-1), and NIX [29]. Fundc-1 is also involved in the regulation of mitochondrial fusion and fission through cooperation with Opa1 and Drp1, respectively, located in the OMM. Interplay with Opa1 also suppresses mitophagy and fission. In contrast, Fundc-1 association with Drp1 triggers these processes [30].

The current knowledge contributes to the theory in which altered mitophagy can be caused by the suppression of fission. Mitochondrial population formed by mitochondrial fission is characterized by reduced membrane potential and decreased levels of the fusion protein Opa1. Lowered Opa1 levels can also participate in dysfunctional mitochondria segregation, which alleviates the detection and removal by mitophagy [31]. Cardiac accumulation of dysfunctional mitochondria occurred without a concomitant increase in mitophagy in response to cardiac deletion of Mfn1 and Mfn2. This allows us to suggest that mitochondrial fusion is a cornerstone in mitochondrial quality control [32]. However, a single genetic deletion of Mfn1 had a slight effect on cardiomyocytes’ mitochondria, while a single Mfn2 deletion led to the accumulation of enlarged mitochondria and an altered autophagosome–lysosome fusion, which is the final step of the autophagic flux. Thus, Mfn2, but not Mfn1, appears to play an essential role in mitochondrial quality control, including mitophagy [33]. Parkin-dependent mitophagy can be promoted by Mfn2 phosphorylation by Pink1, which facilitates the recruitment of parkin to the OMM. Notably, lowered mitochondrial fusion by the suppression of mitofusin limited cardiomyopathy in a Drosophila model of parkin gene deletion, which displayed mitophagy impairment. Parkin deficiency promotes the fusion of dysfunctional mitochondria (not removed by mitophagy) and healthy ones [34]. Additionally, there is interplay between Drp1-mediated fission and non-specific phosphorylation of parkin. This contributes to the protection of normal mitochondria from unnecessary and mistargeted Pink1-Parkin pro-autophagic activity [35]. Notably, Drp1 activation along with increased autophagy was triggered by Bnip3 overexpression in cardiomyocytes, while Drp1 suppression was enough to reverse the effects of Bnip3 overexpression in these cells. In addition, the increased fragmentation caused by Fis1 overexpression promoted mitochondrial dysfunction and enhanced autophagosome formation [36].

## 3. Implications in Cardiovascular Aging

Managing age-associated diseases has become a serious challenge due to increasing life expectancy. Morphological and histological alterations both play roles in cardiac aging. The aged heart is characterized by fibrosis, diastolic dysfunction, and ventricular hypertrophy. During the aging processes, dysfunctional mitochondria accumulation occurred in response to oxidative stress [37]. Mitochondrial permeability transition pore (mPTP) opening was also altered, which resulted in calcium mishandling, the loss of mitochondrial membrane potential, and apoptosis. The association with altered mitochondrial dynamics is also illustrated by the accumulation of giant mitochondria with damaged cristae structures and mtDNA [38]. Studies on animal models revealed the decreased expression of Mfn1/2 in the heart of 25-month-old rats, while Opa1 and Drp1 expression was increased in 36-month-old rats [39].

Most results suggesting that the shift in mitochondrial dynamics towards mitochondrial fusion promotes cell senescence were obtained in in vitro studies. More precisely, the knockdown of Fis1 contributed to increased senescence and resulted in elongated mitochondria in non-cardiac cell lines. Interestingly, the simultaneous depletion of Opa1 reversed cell senescence in Fis1 knockdown cells [40]. Additionally, cell senescence appeared to be enhanced in vitro, as well as Mfn1 expression levels in response to the loss of the mitochondrial E3 ubiquitin ligase MARCH5, an interacting protein of Fis1, Drp1 and Mfn2. These effects were reversed by Drp1 overexpression [41]. In summary, these results contribute to the theory that stimulating mitochondrial fission can decrease aging-associated alterations through mechanisms including the recovery of mitophagy, which is usually decreased in aged tissues. This evidence is supported with several in vivo studies. Physiological cardiac hypertrophy was observed in transgenic mice overexpressing Opa1 at nine months [42]. Cardiac deletion of parkin resulted in fatal cardiomyopathy in mice, while parkin overexpression was shown to have an ability to postpone heart aging. Likewise, a mutation in the Pink phosphorylation site in Mfn2 prevented cardiac metabolism and inhibited mitophagy. Similarly, cardiac impairments associated with aging were shown to be lowered by p53 inhibition via enhancing parkin-mediated mitophagy [43].

Altered mitochondrial fusion is another factor promoting heart aging. Compared to wild-type mice, heterozygous Opa1 knockout mice were shown to have worse cardiac function and fragmented dysfunctional mitochondria while aging [44,45]. Consistently, mice with cardiac deletion of Mfn2 showed reduced left ventricular function as compared to the matched wild type mice at the age of 17 months [46].

Thus, maintaining the balance between mitochondrial fission and fusion could be a more promising strategy to delay cardiac abnormalities related to aging than stimulating or suppressing one of these mechanisms. This is additionally illustrated by the observation of the murine model in which Mfn-mediated fusion and Drp1-mediated fission were blocked in the heart [47]. This model demonstrated a better phenotype compared to the models lacking fusion or fission alone in terms of survival and the development of cardiomyopathy, although impaired mitophagy and the accumulation of senescent mitochondria could still be observed [48].

## 4. Implications in Cardiovascular Disease

Mitochondrial status firmly defines cardiovascular function and disease. Up to 30% of the cardiac cell volume is filled by mitochondria, which generate energy. This is an important element in maintaining cardiac function. Dysfunctional mitochondria promote heart disease development. Aberrant cardiac mitochondrial homeostasis leads to bioenergetic alterations, as reflected in declined cellular adenosine triphosphate (ATP) production, decreased phosphocreatine (PCr), or the PCr/ATP ratio [49]. Additionally, ischemia–reperfusion injury and heart failure are the result of an impaired ATP supply, owing to alterations in mitochondrial calcium transport, resulting in ROS generation and the mitochondrial permeability transition pore (MPTP) opening [50]. In T2DM mouse models, the link between mitochondrial dysfunction and bioenergetics was also observed. Enhanced myocardial oxygen consumption along with decreased cardiac efficiency, which were shown in mouse model, were also reported in obese humans [51].

Cardiac disorders involve different aspects of mitochondrial quality control. Thus, cardiomyopathy, which is the result of the loss of Dars2 (mitochondrial aspartyl-tRNA synthetase 2), presents impaired mitochondrial proteostasis and mitochondrial oxidative phosphorylation (OXPHOS) system in the heart and the induction of the UPR^mt^ [52]. It was reported that mitochondrial dynamics are essential due to the discovery that the alteration of the fusion proteins mitofusin 1 and 2 (MFN1/2) in mice and flies or dysregulated proteolysis of the IMM fusion protein optic atrophy protein 1 (OPA1) resulted in dilated cardiomyopathy and heart failure [53].

The development of left ventricle hypertrophy was shown to be suppressed in the presence of a dominant-negative mutation in dynamin-related protein 1 (DRP1), which is able to stimulate mitochondrial fission under normal conditions [8,54]. The mitochondrial dynamics are also crucial for the dilated cardiomyopathy (DCM) development. An increased mitochondrial emission of ROS along with disrupted mitochondrial morphology occurred alongside decreased MFN1 levels in humans. The role of this quality control pathway in cardiomyopathy has also been illustrated in mouse models of DCM [55].

There are numerous associations between cardiac function and molecules, which are important for mitochondrial function, including sirtuins and PGC-1α. The family of energy-sensing sirtuins appeared to have cardioprotective attributes, while one member (SIRT4) altered cardiac functioning, potentially predisposing mice to cardiac hypertrophy. An altered mitochondrial biogenesis contributes to mitochondrial cardiomyopathies [56].

Peripheral artery disease (PAD) is among the most prevalent causes of cardiovascular morbidity resulting in a decreased skeletal muscle oxidative capacity. It was shown in numerous experiments that PAD develops from both an altered blood flow and a disrupted mitochondrial respiratory capacity and quality [57]. This has provided a significant advantage for linking mitochondrial function to metabolic health, as muscle biopsies are readily available in PAD compared to other diseases where the tissue of interest is not commonly sampled, such as the liver, brain, or heart [58].

## 5. Mitochondrial Dynamics in Atherosclerosis and Stroke

The pathogenesis of atherosclerosis remains a complex combination of various processes, combining inflammation, endothelial dysfunction, lipid metabolism impairments, and others. The hallmark of atherogenesis is the formation of plaques, which narrow the artery. These plaques consist of modified lipid particles, inflammatory cells, and various cellular and non-cellular elements. Mitochondrial pathologies have been shown to be another important player in atherogenesis [59].

Both in humans and in animal models, the expression of Mfn2 appeared to be decreased in thrombi. Thus, the overexpression of Mfn2 was shown to lower atherosclerotic lesions in a rabbit model and to suppress neointimal formation in rat balloon-injured arteries [60,61].

The implication of mitochondrial dynamics in endothelial dysfunction was investigated in endothelial cells obtained from diabetic patients with decreased vascular function. In these cells, fragmented mitochondria and overexpression of Fis1 were observed. This effect was rescued in vitro by fission inhibition [62]. When treated with retinol binding protein 4 (RBP4), endothelial cells exhibited decreased fusion along with enhanced fission. RBP4 is an adipokine usually found at high levels in the blood of patients with metabolic syndrome [63].

Endothelial cells are not the only cells that proliferate and migrate to the site of the atherosclerotic plaque formation. Vascular smooth muscle cells (VSMCs) are also involved in this process. Being activated by PDGF (platelet-derived growth factor) appeared to be associated with reduced Mfn2, while fission inhibition decreased VSMC proliferation [64]. The suppression of Drp1 was shown to significantly decrease VSMC proliferation and migration, as well as the formation of neointima. These data were obtained in an ex vivo aortic ring assay in a model of rat carotid artery balloon injury. It was also observed that the suppression of Drp1 can decrease endothelial dysfunction and atherosclerosis in apolipoprotein E (ApoE) knockout diabetic mice and, moreover, reduce the calcification of smooth muscle cells caused by oxidative stress [65]. In summary, these results allow us to suggest that fission inhibition or fusion stimulation are potential strategies by which to slow down atherogenesis.

Mitochondrial disfunction is a part of ischemic stroke pathophysiology. Thus, mitophagy recovery appeared to suppress the predisposition to stroke in a model of essential hypertension and spontaneous stroke. Mitochondrial dynamics are an important mechanism of cell death and survival during cerebral ischemia due to the fact that mitochondrial fission is an early event preceding neuronal loss in a murine middle cerebral artery occlusion (MCAO) I/R model [66,67]. Similarly, the infarct volume appeared to be decreased in MCAO models in response to the inhibition of Drp1, which also amended mitochondrial function and decreased mitochondrial fragmentation. In a permanent MCAO model, downregulation of Mfn2 was observed. Thus, elevated mitochondrial fission can be suggested to be an ischemic stroke contributor. Of course, further studies are needed to determine the role of mitochondrial dynamics in cerebral vascular cells and neuronal death [68]. Data on various proteins participating in mitochondrial dynamic effects on various cardiac impairments are summarized in Table 2.

## 6. Future Directions and Perspectives

Despite the improved understanding of the crucial role of mitochondrial dynamics in various cardiovascular alterations, there are very few therapeutic strategies. Targeting mitochondria and their dynamics seems to be beneficial and thus has attracted the attention of investigators. It seems that targeting mitochondria with newly developed therapeutics may alleviate the consequences of ischemic stroke, and this also has potential in the treatment of cerebral ischemia [69]. A neuroprotective effect was demonstrated for pharmacological factors or genetic interventions targeting mitochondrial dynamics and quality control in preclinical studies. However, in clinical practice, targeting mitochondria remains challenging [70].

Recent studies have reported that restocking healthy mitochondria and utilizing the damaged ones can be used to treat hypoxia/ischemia-related diseases. Various extracellular stimuli can launch “help-me” signaling in the damaged mitochondria, leading to the recruitment of adjacent cells to rescue the impaired cells. This is of high importance for CNS because the neuronal synapses and dendrites contain many mitochondria [71].

Another approach is based on stem cells, which appeared to have a mitochondrial-protective effect in numerous pre-clinical settings. It seems that stem cells can transfer mitochondria to the impaired cells via tunneling nanotubes, extracellular vesicles, or by cellular fusion, helping to revive cell energetics in the recipient cell. Mitochondrial transplantation was shown to be successful in the patients suffering from myocardial IR injury, but it is still far from being implemented in clinical practice [72].

In rodents, MDIVI-1, the chemical inhibitor of DNM1L, was shown to mediate cardioprotective effects in cardiomyopathy and ischemia-reperfusion injury models. However, the specificity of its action is doubtful. Additionally, similar observations were made for other DNM1L inhibitors such as P110 and dynasore. Another developed agent is a cell-permeant peptide enabling MFN2-dependent mitochondrial fusion, but there are no data, as far as we know, of clinical trials for any of these approaches [73].

Interesting observations were made in studies on dogs with coronary microembolization-induced chronic heart failure. It was shown that the daily administration of elamipretide in the form of subcutaneous injections for three months improved left ventricular (LV) systolic function and prevented progressive LV dilation without affecting heart rate, blood pressure, or systemic vascular resistance. Elamipretide also improved mitochondrial function, which was illustrated by decreased ROS production, the normalization of membrane potential, and an improved maximum rate of ATP synthesis. In LV tissue from these dogs, the prolonged elamipretide administration reversed the dysregulation of mitochondrial fission and fusion proteins and Pparg coactivator 1 alpha (PGC-1α) protein levels, supporting the observations of improved mitochondrial respiration, an improved maximum rate of ATP synthesis, and improved LV function. Additionally, the dysregulation in cardiolipin and the determinants of cardiolipin synthesis and remodeling, including cardiolipin synthase-1, TAZ-1, and acyl-CoA:lysocardiolipin acyltransferase-1, were normalized after three months of therapy [74].

## 7. Conclusions

Mitochondria play an important role both in the normal functioning of cells and in the development of various pathologies. The results of numerous studies leave no room for doubt regarding the importance of mitochondria in the cardiovascular system. Mitochondrial dynamics and shifts in the balance between fusion and fission deserve special attention.

Initially, scientists relied on stimulating or inhibiting only one of the two processes, but over time, it has become obvious that maintaining a balance between mitochondrial fission and fusion can be a more promising strategy by which to delay cardiac abnormalities related to aging than stimulating or suppressing one of these mechanisms. This balance is influenced by the modulation of proteins that control fusion and fission.

All of this gives scientists a reason to continue paying attention to these proteins as potential targets for the prevention and, possibly, treatment of cardiovascular diseases. We believe that mitochondrial targeting is a promising field of investigation. A range of preclinical studies have shown its beneficial potential. However, the exact mechanisms of the actions of such drugs are doubtful. This makes the translation to clinical practice almost impossible. To overcome these obstacles, further research and trials are certainly needed.

## Figures and Tables

**Table 2:** Summary of proteins associated with mitochondrial dynamic effects on various cardiac impairments.

Protein	Disease/Condition	Model	Reference
Knockdown of *Fis1*	Increased senescence, elongated mitochondria in non-cardiac cell lines	In vitro	[40]
Simultaneous depletion of *Opa1*	Reversed the knockdown of Fis1 effects	In vitro	[40]
Enhanced *Mfn1* expression	Increased senescence	In vitro	[41]
Overexpression of *Opa1*	Physiological cardiac hypertrophy	Transgenic mice	[42]
Cardiac deletion of *parkin*	Fatal cardiomyopathy	Mice	[43]
Overexpression of *parkin*	Delay of the heart aging	Mice	[43]
Inhibition of p53	Lowering of age-associated cardiac impairments	Mice	[43]
Knockout of *Opa1*	Worse cardiac function and fragmented dysfunctional mitochondria while aging	Heterozygous mice	[44,45]
Cardiac deletion of *Mfn2*	Reduced left ventricular function	Mice	[46]
Loss of Dars2	Cardiomyopathy	Mice	[52]
Alteration of MFN1/2	Dilated cardiomyopathy and heart failure	Mice	[53]
Dysregulated proteolysis of Opa1	Dilated cardiomyopathy and heart failure	Mice	[53]
Dominant-negative mutation in *DRP1*	The development of left ventricle hypertrophy inhibition	In vitro	[54]
Overexpression of *Mfn2*	Lowering of atherosclerotic lesions	Rabbits	[60]
Overexpression of *Mfn2*	Suppression of neointimal formation	Rat balloon-injured arteries	[61]
Overexpression of *Fis1*	Fragmented mitochondria	Endothelial cells obtained from diabetic patients with decreased vascular function	[62]
Treatment with RBP4	Decreased fusion and enhanced fission	Endothelial cells	[63]
Suppression of Drp1	Decreased VSMC proliferation and migration and formation of neointima	Ex vivo aortic ring assay in a model of rat carotid artery balloon injury	[65]
Suppression of Drp1	Decreased endothelial dysfunction and atherosclerosis, reduced calcification of smooth muscle cells caused by oxidative stress	(*ApoE*) knockout diabetic mice	[65]
Inhibition of Drp1	Decreased infarct volume	MCAO model	[68]
Downregulation of *Mfn2*	Elevated mitochondrial fission	Permanent MCAO model	[68]

Mfn1—mitofusin1; Mfn2—mitoofusin 2; Opa1—optic atrophy protein 1; Parl—presenilin-associated rhomboid-like protein; Oma1—mitochondrial metalloendopeptidase Oma1; Yme1L1—ATP-dependent zinc metalloprotease YME1 Like 1 ATPase; Afg3l1—mAAA protease complex ATPase family gene-3 yeast-like-1; Drp1—dynamin-related protein 1; Fis1 - mitochondrial fission protein 1; Mdv1—mitochondrial division protein 1; Mff—mitochondrial fission factor; SNX9—sorting nexin 9; RBP4—retinol binding protein 4; Dars2—mitochondrial aspartyl-tRNA synthetase 2; MCAO—middle cerebral artery occlusion.

## Data Availability

Not applicable.
